# Decoding CTCs in osteosarcoma: the molecular journey from initial tumor to metastasis

**DOI:** 10.1038/s41598-026-47094-5

**Published:** 2026-04-03

**Authors:** Alessandro Di Gangi, Mariangela Morelli, Edoardo Ipponi, Fabrizio Catena, Luca Coccoli, Raffaele Gaeta, Paolo Aretini, Michele Menicagli, Alessandro Franchi, Gaia Palmini, Gabriella Casazza, Lorenzo Andreani, Teresa Iantomasi, Maria Luisa Brandi, Chiara Maria Mazzanti, Francesca Lessi

**Affiliations:** 1 Health Science Interdisciplinary Center, Sant’Anna School of Advanced Studies, Pisa, Italy; 2 Fondazione Pisana per la Scienza ONLUS, via Ferruccio Giovannini, 13, San Giuliano Terme, 56017 Pisa, PI Italy; 3 Department of Orthopedics and Trauma Surgery, University of Pisa, Pisa, Italy; 4 Pediatric Onco-Hematology Unit, S. Chiara Hospital, AOUP, Pisa, Italy; 5 Section of Pathology, Department of Translational Research and New Technologies in Medicine and Surgery, University of Pisa, Pisa, Italy; 6 Department of Experimental and Clinical Biomedical Sciences “Mario Serio” (DSBSC), University of Florence, Florence, Italy

**Keywords:** Circulating tumor cells, Osteosarcoma, Liquid biopsy, Copy number alterations, Mesenchymal-to-epithelial transition, Whole-exome sequencing, Biomarkers, Cancer, Oncology

## Abstract

Metastatic relapse remains the main cause of treatment failure in pediatric osteosarcoma (OS), as conventional imaging often misses minimal residual or low-burden disease. Circulating tumor cells (CTCs) represent a promising non-invasive biomarker for monitoring tumor progression and heterogeneity. We developed an integrated workflow to isolate, enrich, and molecularly characterize CTCs from six pediatric OS patients. Peripheral blood mononuclear cells were enriched using the Parsortix system. CTCs were classified by immunofluorescence using epithelial (EpCAM, CK8/18/19) and mesenchymal (Vimentin, TWIST1) markers. Single cells were isolated via DEPArray, followed by whole-genome amplification and low-pass sequencing to assess copy number alterations (CNAs). In one patient, whole-exome sequencing (WES) was performed on bulk CTCs, primary tumor, and metastatic tissue to confirm tumor origin and identify shared somatic mutations. Across the cohort, 908 CTCs were identified, predominantly mesenchymal, with a smaller epithelial subset showing higher genomic complexity and CNA burden. WES confirmed overlapping mutations and CNAs among CTCs, primary, and metastatic tumors. Epithelial CTCs exhibited greater genomic instability than mesenchymal cells. Phenotype correlations with treatment response suggested potential links to histological necrosis. This study provides the first comprehensive phenotypic and genomic characterization of CTCs in pediatric OS, confirming their neoplastic nature and molecular heterogeneity. These findings support CTC-based monitoring as a minimally invasive tool for guiding personalized therapy and improving disease surveillance in high-risk OS patients.

**Supplementary Information:**

The online version contains supplementary material available at 10.1038/s41598-026-47094-5.

## Introduction

Bone tumors account for almost 4% of childhood cancers and, among them, osteosarcoma (OS) represents the most common malignant bone tumor with, an incidence in Europe ranging 3–5 per million in males and 2–4 per million in females^1^. Multiple relapse/refractory disease and lung metastasis are the most important determinants of patients outcomes^2,3^. The histological degree of necrosis after the neoadjuvant chemotherapy is crucial for identifying patients at risk of recurrence^4^ and current risk-adapted treatment strategies are mainly based on this parameter^5^. Although slight differences exist among clinical protocols, lung metastatic disease at diagnosis is usually defined as A) 1 or more pulmonary/pleural nodules ≥ 1 cm, or B) 3 or more nodules ≥ 0.5 cm in maximum diameter, while fewer or smaller lesions are classified as “possible” metastasis^5^. The complex interplay between tumor cells and their microenvironment drives the “seed and soil” phenomenon that ultimately led to lung metastasis^6–8^, in which mesenchymal stem cells play a pivotal role^9^. The process of tumor cell dissemination is highly complex, involving various components of the tumor microenvironment, such as blood vessels and immune cells^7^. However, the precise mechanisms underlying the metastatic spread in OS remain poorly understood, emphasizing the need for further research in this area. Beyond these biological aspects, a possible explanation for the inconsistent detection of metastatic lesions lies in the limited sensitivity of current imaging techniques. These limitations hinder the reliable recognition of small lesions and prevent the identification of patients with low-burden metastatic disease. Therefore, novel biomarkers are urgently needed and liquid biopsy approaches hold the potential to detect disseminated disease even before it becomes clinically evident. In parallel, recent whole-exome sequencing analyses have provided a comprehensive view of the molecular landscape of high-grade OS, unveiling recurrent alterations linked to genomic instability, tumour progression, and therapeutic resistance^10^. These molecular insights form the basis for the search for minimally invasive biomarkers capable of reflecting the tumour’s genetic heterogeneity and its clinical behaviour.

Among the available liquid biopsy strategies, cell-free DNA (cfDNA) and circulating tumor DNA (ctDNA) are increasingly studied as dynamic markers for treatment response and patient stratification due to the robust assays available^11,12^. However, correlating cfDNA/ctDNA levels with metastatic disease remains challenging; in a recent study, even patients with metastatic OS were found to be ctDNA-negative at diagnosis^13^. Consequently, the development of reliable biomarkers capable of accurately predicting recurrence and metastatic progression is highly warranted. In this context, circulating tumor cells (CTCs) represent the cornerstone of the liquid biopsy paradigm, providing a dynamic and easily obtainable window into tumor characteristics without directly sampling the tumor mass. The complexity of CTC analysis in OS lies in two main aspects: the lack of specific markers and the mesenchymal nature of the neoplasm^14^, which differentiates it from other cancers, such as lung or breast carcinoma, where CTCs are typically identified through epithelial markers due to the epithelial–mesenchymal transition (EMT) process^15^. Nevertheless, the homing of CTCs to distant metastatic niches remains a core phenomenon that attracted the attention of researchers for decades^16^. In this regard, a marker-dependent system called CellSearch is one of the most applied and relies on the selection and identification of cells expressing the epithelial marker EpCAM^17,18^. For some authors, this approach is inherently limited in OS, where CTCs arise from a tumor with mesenchymal phenotype. However it is true that epithelial-like CTCs have also been identified in OS patients^19^, likely due to the process of mesenchymal-to-epithelial transition, the reverse phenomenon of EMT where mesenchymal cells, typically more motile and invasive, regain epithelial traits such as cell adhesion and intercellular junctions^20^. In this work, we developed a protocol to isolate CTCs through a physical-based enrichment followed by marker-dependent selection and subsequent validation through genomic analysis, in order to verify tumoral hallmarks within the isolated CTCs and to investigate possible clinical correlations in pediatric patients affected by OS. To further strengthen the analysis, we have also performed an exploratory evaluation on pooled (bulk) CTC populations (BCTCs) rather than single cells, allowing us to capture a broader and more representative view of the tumor’s genetic heterogeneity and clinically relevant alteration.

## Results

### Study population

We enrolled 6 children/AYAs affected by OS; 1 patient missed the blood sampling at the time of the surgical treatment (OS2). Therefore, only 5 pre- and post-neoadjuvant treatment pairs were available for analysis. Four healthy controls were included in the study. Descriptive statistics for the OS patients are presented in Table 1 and additional details on patients’ clinical course are presented in Supplementary.

**Table 1:** Study population.

Characteristic	Count (%)	Characteristic	Count (%)
Age at diagnosis, median years (IQR)	12.5 (7.0)	AJCC staging—count (%)	
Male, n (%)	4 (66.7)	IIA	2 (33.3)
Site, n (%)		IIB	3 (50.0)
Femur	3 (50.0)	III	1 (16.7)
Radius	1 (16.7)	Histology—count (%)	
Tibia	1 (16.7)	Osteoblastic	5 (83.3)
Humerus	1 (16.7)	Small cells	1 (16.7)
Position, n (%)		Percentage of histological necrosis, median (IQR)	80 (20)
Proximal	2 (33.3)	Risk group—count (%)	
Distal	4 (66.7)	*GR*	1 (16.7)
Side, n (%)		*PR*	5 (83.3)
Right	4 (66.7)	SUVmax at diagnosis, median (IQR)	13.3 (18.8)
Left	2 (33.3)	SUVmax before local treatment, median (IQR)	3.7 (7.6)
Presence of lung nodules at the diagnosis, n(%)	3 (50.0)	∆SUVmax, median (IQR)	5.3 (21.0)
Possible metastasis*	2 (66.7)	Adjuvant protocol, n (%)	
Metastatic nodules*	1 (33.3)	MAP	4 (66.7)
Enneking staging—count (%)		HD-IFO	2 (33.3)
IIA	3 (50.0)	Best response—count (%)	
IIB	2 (33.3)	NED	5 (83.3)
III	1 (16.7)	AWD	1 (16.7)

Six patients were effectively enrolled. *At the time of diagnosis, lung nodules were defined as metastatic if metastatic nodules were defined as A) 1 or more pulmonary/pleural nodule(s) ≥ 1 cm or B) 3 or more nodules ≥ 0.5 cm as maximum diameter C) when those criteria were not met, patients were classified as metastatic according to the clinical suspicious lead by an expert radiologists (ex. a calcified nodule > 0.5 cm but < 1 cm). All other findings were categorized as possible metastasis. GR: good responders, HD-IFO: high-dose ifosfamide, IQR: inter quartile range, MAP: methotrexate-adryamicin-cisplatin, PR: poor responders, SUV: Standard Uptake Value.

The median age at diagnosis was 12.5 (± 7.0) years, male gender was slightly more represented. All the males with distal femurs involvement were affected on the right side while the only female with this localization had a left sided tumor.The median SUVmax at the diagnosis was 13.3 (IQR 18.8, range 6.5–25.4). Notably, only OS4 was defined as metastatic at the diagnosis while pulmonary micronodules without a stringent metastatic definition were observed in other 2 patients. At the pre-surgical reassessment, OS2, OS3 and OS12 did not demonstrate the presence of micronodules but OS12 was classified as widely metastatic.

The only GR in our cohort was OS17 with a necrosis of 99% while OS12 had an undesirable necrosis of 45% (very-poor responder). The median necrosis was 80% (IQR 20%, range 45–99%). SUVmax largely decreased without statistical significance (n = 6, Wilcoxon paired-signed-rank test; z = 1.57 *p* = 0.11) at the time of the pre-surgical reassessment. The only exception was OS12 where an increase related to the presence of a chemo-refractory disease was observed. The median SUVmax at the reassessment was 3.7 (IQR 7.6 range 3.2–14.4). The median change of the SUVmax between diagnosis and reassessment (∆SUVmax) was 5.3 (IQR 21.0 range − 4.8 to 22.1). The greater decrease in ∆SUVmax was observed in OS17.

### Proof-of-concept: OS4 enriched blood fraction retains osteosarcoma-derived cells

**Fig. 1:**
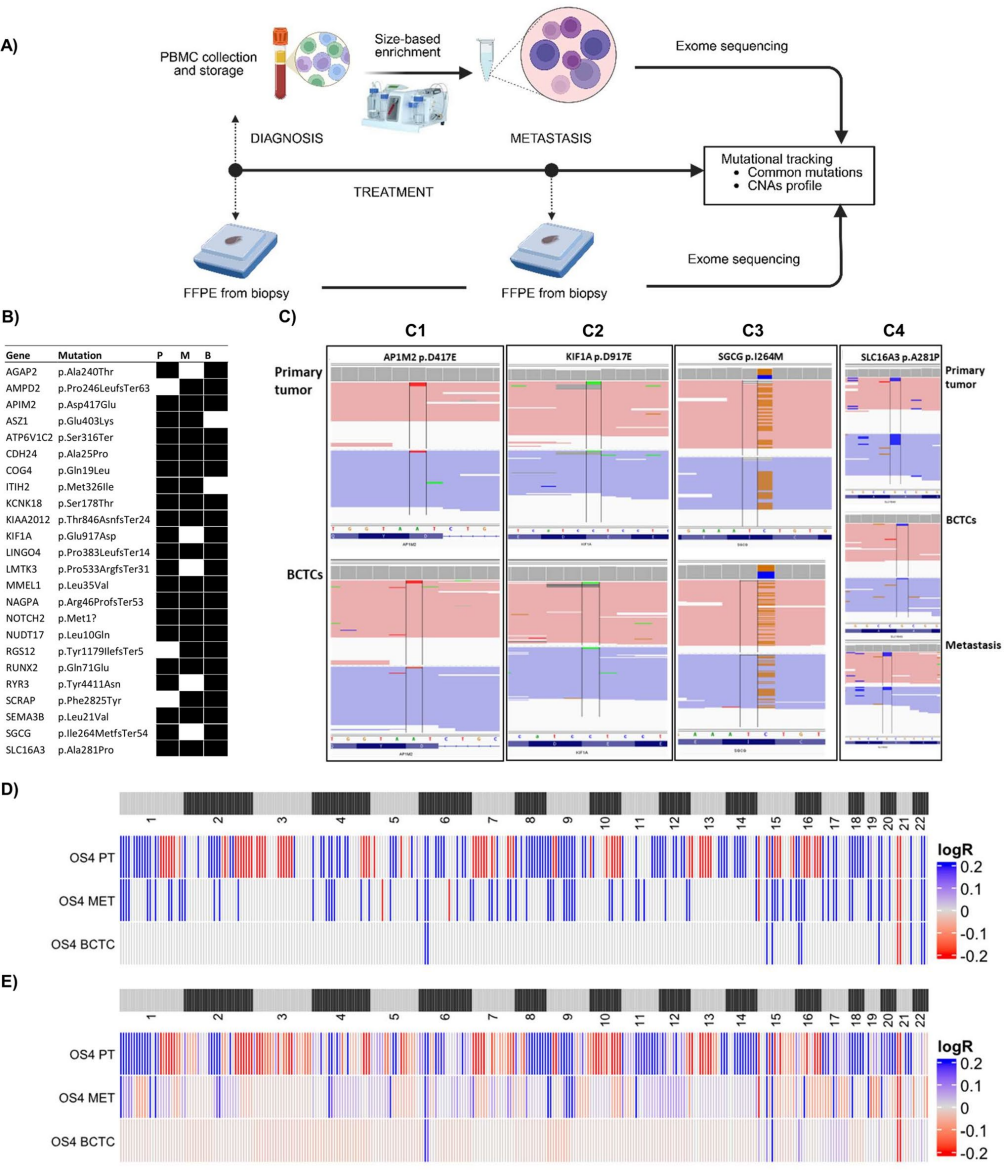
Proof-of-concept study to demonstrate the neoplastic nature of BCTCs. (**A**) Schematic workflow of OS4 sample collection and analysis. PBMC is collected and stored, and circulating tumor cells are enriched using a size-based method before exome sequencing. FFPE tumor tissues are obtained at diagnosis and at local metastasis, followed by exome sequencing. Comparative analysis enables mutational tracking, including identification of common mutations and CNAs. (**B**) Scheme of patient OS4’s somatic mutations. The figure illustrates the somatic mutations detected in this patient that are shared among the primary tumor, the recurrences, and the bulk CTCs. B: BCTCs. (**C**) IGV visualization showed SNPs in different genes in common between the samples: (C1) The IGV screenshot of APIM2 gene variation p.D417E in primary tumor (above) and in BCTCs (below). (C2) The IGV screenshot of KIF1A gene variation D917E. (C3) The IGV screenshot of SGCG gene variation p.I264M. (C4) The IGV screenshot of SLC16A3 gene variation p.A281P in primary tumor (above), in BCTCs (in the middle) and in metastasis tissue (below). (**D**,**E**) Genome-wide chromosome arm profile heatmap for matched primary tumor, metastasis and BCTCs with CNApp tool. On the left lane, the CNA profiles of the primary tumor, in the middle lane the metastasis and on the right lane the BCTCs are shown. The chromosomal amplifications are shown in red, and the deletions in blue. In (**D**) the all CNAs profiles and in (**E**) the focal CNAs. B/BCTCs: pooled (bulk) CTCs population, CNAs: copy number alterations; IGV: Integrative genome viewer; FFPE: Formalin-Fixed Paraffin-Embedded; M: metastasis; P: primary tumor; PBMC: peripheral blood mononuclear cells, SNPs: single nucleotide polymorphisms.

We confirmed the presence of neoplastic elements within the BCTCs product trought the comparative WES analysis of the primary tumor, the lung metastasis and the BCTCs derived from one OS patient (OS4). Study design and results for OS4 patient are presented in Fig. 1. We obtained 73, 47 and 49 confirmed variants in primary tumor, metastasis and BCTCs samples respectively.

Common variants are listed in Fig. 1B and presented in panel C torught the IGV visualization. Panel C1 illustrates the p.D417E variants of APIM2 gene, consistently identified in both the primary tumor (C1 upper) and the corresponding BCTCs sample (C1 lower), indicating its presence early in the tumor development and its persistence in circulating cells. Similarly the KIF1A p.D917E variant was also shared by primary tumor and BCTCs (Panel C2). Panel C3 highlights the p.I264M variant within the SGCG gene, another recurrent alteration observed among primary tumor and BCTCs. Furthermore, panel C4 provides an example of a variation found across all three distinct samples: the p.A281P alteration in the SLC16A3 gene. This particular variant was identified in the primary tumor (C4 upper), in the BCTCs fraction (C4 middle), and notably, also in the lung metastasis (C4 lower). The presence of these specific variants in APIM2, KIF1A, SGCG, and SLC16A3 across primary tumor, BCTC, and/or lung metastasis strongly supports their potential role as stable genetic markers traceable throughout disease progression and dissemination.

CNAs analysis was conducted from WES data and the results are shown in panel D) and E). In this landmark experiment, a clear chromosomal alteration signature was observed, specifically, BCTCs consistently displayed a CNA pattern shared with the primary tumor and with metastasis, highlighting how the CTCs reflected the genomic profile of the tumor.

Despite being a proof-of-concept experiment limited to a single case in our cohort, these findings confirm the presence of tumor cells within BCTCs. The fact that their genomic profile matches the metastatic tissue suggests they potentially contribute to the dissemination of the primary neoplasia.

### Enumeration of E-CTCs could be linked to histological necrosis

Globally, we identified 908 CTCs from our cohort of patients (PRE n = 6, cells = 437; POST n = 5, cells = 471) with a median of 60 CTCs/patient PRE (IQR 123) and 38 CTCs/patient POST (IQR 175). When analyzed by phenotype, we successfully isolated a median of 4 E-CTCs/patient PRE (IQR 15), 48 M-CTCs/patient PRE (IQR 127), 1 E-CTC/patient POST (IQR 9) and 40 M-CTCs/patient POST (IQR 175). Notably, only 2 CTCs with a hybrid phenotype (M/E) were observed in one patient (OS2) pre-treatment. We could not identify E-CTCs or M-CTCs in 4 healthy controls while Hoechst -positive cells were present in the BCTCs.

**Fig. 2:**
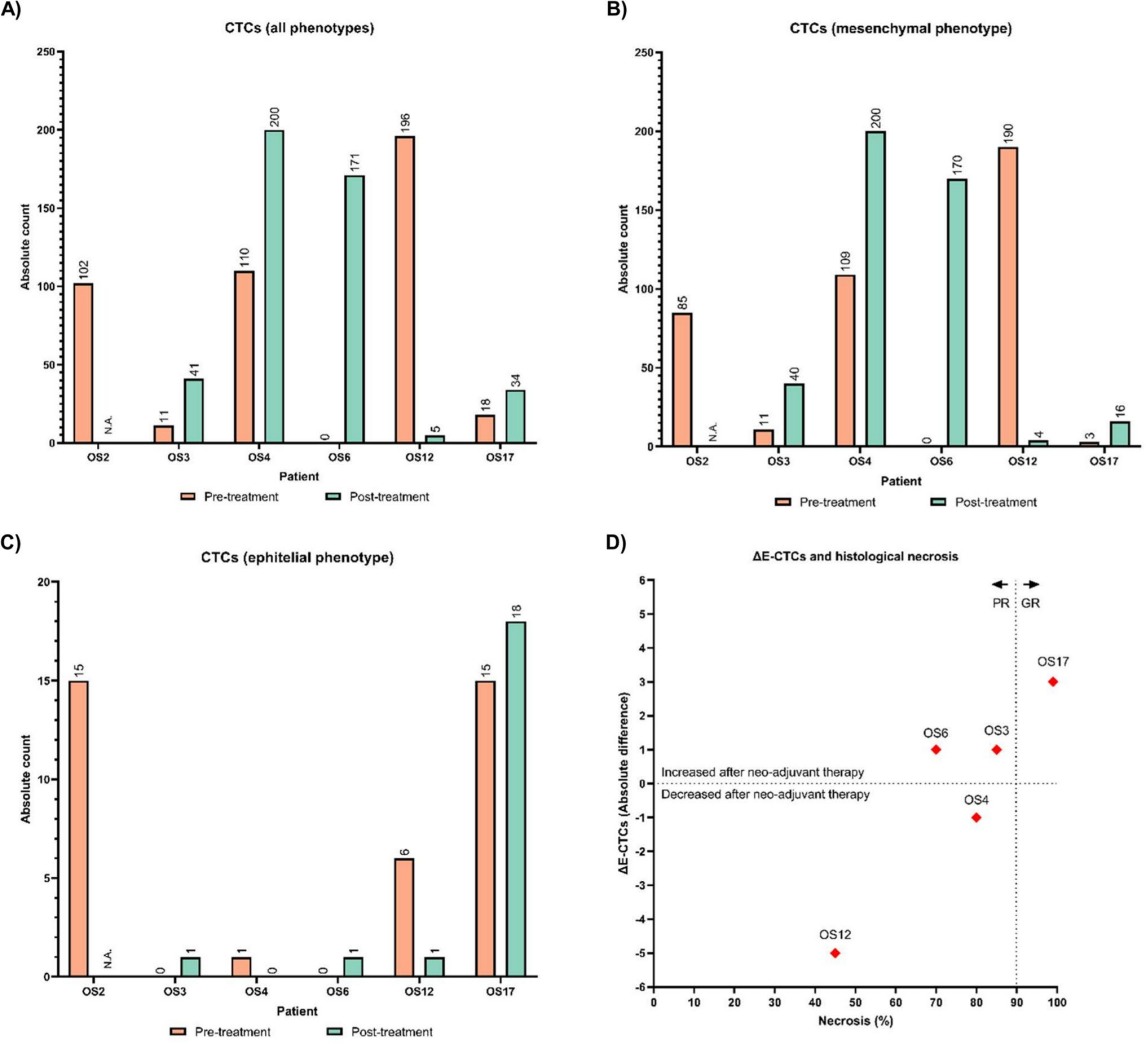
CTCs enumeration. (**A**) Total CTC counts PRE/POST patient. With the exception of OS12, an increase in CTCs was found in POST samples. POST sample was not collected for OS2. (**B**) Epithelial CTCs. Although more rare, more than 1 epithelial CTCs were identified in 50% (3/6) of PRE samples and 20% (1/5) in POST samples. (**C**) Mesenchymal CTCs. The majority of the identified CTCs exhibit a mesenchymal phenotype. Mesenchymal CTCs were increased in 80% (4/5) POST samples. Of note, 2 CTCs with the simultaneous expression of mesenchymal and epithelial markers were identified in OS2 (not shown). (**D**) Correlation between tumor necrosis (%) after neoadjuvant treatment and variation in E-CTCs (ΔE-CTCs). Each dot represents an individual patient. The vertical dotted line at 90% necrosis separates PR patients from GR ones based on histological criteria. The horizontal dotted line indicates no change in E-CTC count. While not statistically significant (r = 0.82, *p* = 0.133), considering the limited number of observation and the dispersion of the other data, this correlation could be proposed for subsequent investigational endpoints in larger cohorts of patients. CTCs: circulating tumor cells; E: epithelial-phenotype; GR: good responders; M: mesenchymal-phenotype; POST: after neoadjuvant therapy (before the surgery); PR: poor responders; PRE: pre-treatment.

No significant difference in the total number of CTCs PRE and POST treatment was observed (n = 5, cellsPRE = 335, cellsPOST = 471, Wilcoxon signed rank, Z = 0.11, *p* = 0.91) nor in E-CTC (n = 5, cellsPRE = 22, cellsPOST = 21, Wilcoxon signed rank, Z = 0.14, *p* = 0.89) or M-CTCs (n = 5, cellsPRE = 313, cellsPOST = 430, Wilcoxon signed rank, Z = 0.67, *p* = 0.50). In general, it was not possible to demonstrate a trend at patient level, with some patients demonstrating an increase in the number of CTCs or subtypes while others remained stable or decreased (Fig. 2).

No significant correlations were found between CTCs enumeration and clinicopathological parameters (supplementary material). Despite that, it is worth mentioning the correlation between the percentage of necrosis and the ∆E-CTCs, namely the difference of the POST and PRE CTCs count (n = 5, Spearmann rho = 0.82; 95% CI − 0.22 to 0.99, *p* = 0.1333). Intriguingly, while the result should be considered exploratory in the light of the limited sample size, patients with higher tumor necrosis following neoadjuvant therapy exhibited an increase in the number of E-CTCs, while those with lower necrosis more often showed a reduction (Fig. 2). Notably, patient OS17, who was the only GR (necrosis ≥ 90%), showed a clear increase in E-CTCs after treatment. In contrast, patient OS12, who had metastatic disease at diagnosis and demonstrated a very poor response to neo-adjuvant chemotherapy (necrosis < 50%), exhibited the most significant decrease in E-CTC count. These findings could reveal an apparent discordance between local tumor response and the expected systemic tumor cell dynamics.

### CNAs are a complementary hallmark for CTCs

Following initial identification, we used the DEPArray platform to isolate individual cells for genomic analysis. Of the 908 CTCs identified, we successfully performed whole-genome amplification on 5 E-CTCs from two patients, 29 M-CTCs from six patients, and 34 Hoechst positive cells from six patients.

**Fig. 3:**
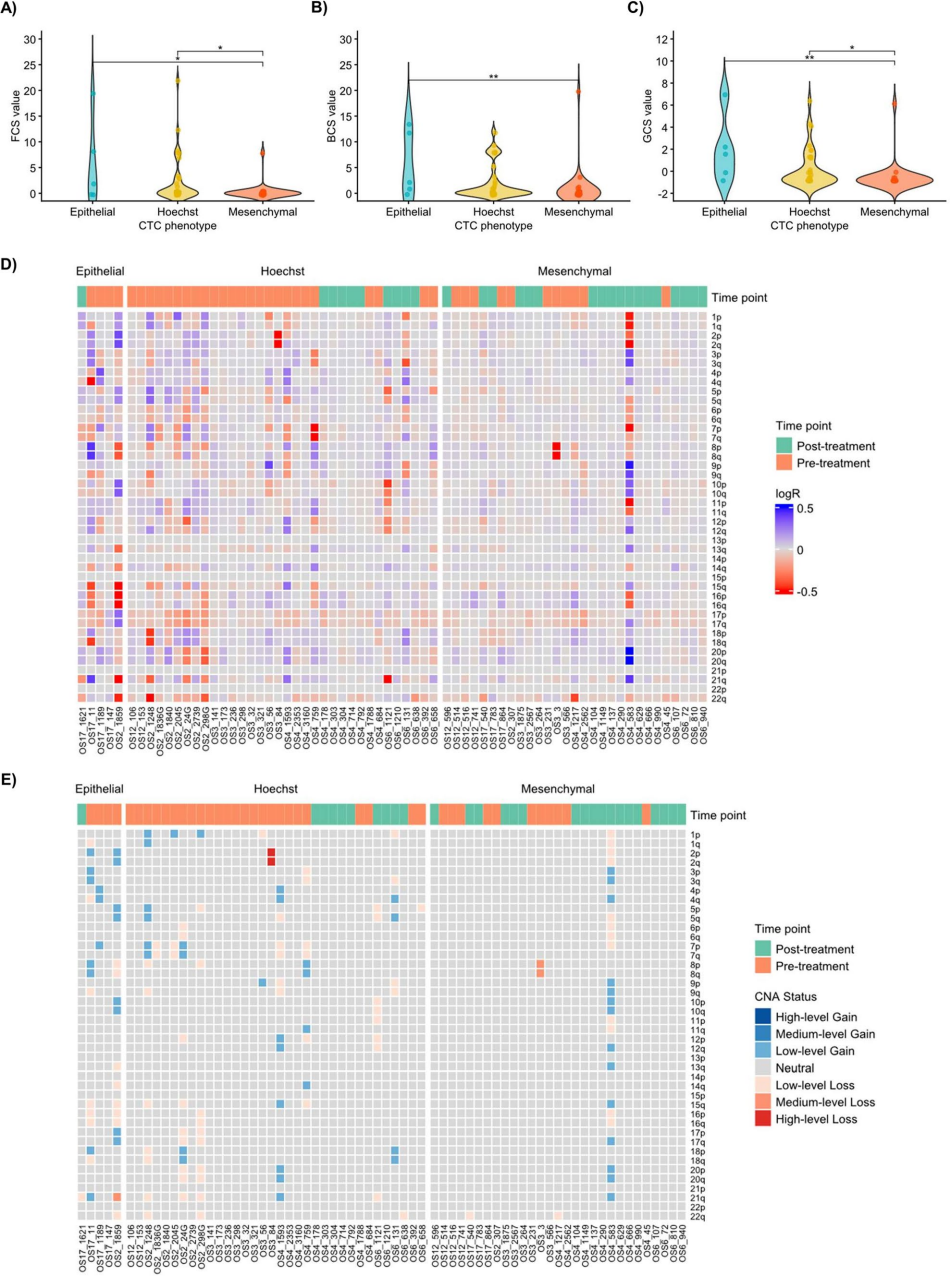
CNAs profiles of isolated CTCs. A) B) and C) showed the violin plot of broad, focal and global CNA scores (BCS, FCS and GCS, respectively) calculated with CNapp. Globally, differences were reported for FCS, BCS and GCS values among different CTCs groups (*p* = 0.00279, *p* = 0.00338 and *p* = 0.00155 respectively) (**A**) FCS values significantly differ from E-CTCs and M-CTCS (*p* = 0.01) and between M-CTCs and Hoechst + cells (*p* = 0.02) while no difference was observed between E-CTCs and Hoechst + cells. (**B**) BCS values were significantly higher in E-CTCs when compared to M-CTCs (*p* = 0.007) but no difference was observed among other post-hoc tests. (**C**) similarly to FCS, GCS is significantly lower in M-CTCs when compared to E-CTCs (*p* = 0.004) and Hoechst + cells (*p* = 0.032) while no other difference was observed. CNAs profiles of isolated single cells are depicted in (**D**,**E**). Unique IDs of isolated cells are shown and the time point of sampling is represented by the color bar on the top of the heatmap (green for pre-treatment and orange for post-treatment samples). Chromosome 19 was omitted from the analysis because it is a common artifact in WGA. Neutral alterations were defined within a range of logR between − 0.2 and + 0.2, otherwise they were defined as losses (< − 0.2) or gains (> 0.2). In (**D**) values were capped at 0.5 that corresponds approximately to the acquisition or loss of an additional copy of the region. In (**E**) CNAs were classified according to their level of alteration into low-level gains (logR 0.2 to 0.58), medium-level gains (logR 0.58 to 1) and high-level gains (logR > 1), low-level losses (logR − 0.2 to − 1), medium-level losses (logR − 1 to − 1.75) and high-level losses (logR < − 1.75). All E-CTCs, with the exception of one, demonstrated CNAs, while M-CTCs presented only minor alterations or none. Surprisingly, several Hoechst cells demonstrated CNAs. BCS: broad CNA score; CNAs: copy number alterations; CTCs: circulating tumor cells; E: epithelial phenotype; FCS: focal CNA score, GCS: global CNA score; M-CTCs: mesenchymal phenotype *p < 0.05 **p < 0.01.

Genomes of the three different different CTC phenotypes were different in term of FCS (H = 11.7609, df = 2, *p* = 0.00279) BCS (H = 1.38222, df = 2, *p* = 0.00338) and GCS (H = 12.9415, df = 2, *p* = 0.00155) as reported in Fig. 3A–C and supplementary material. Dunn post-hoc analysis with Bonferroni correction showed a significant higher FCS for E-CTCs (z = 2.830551, p-adj = 0.0139), BCS (z = − 3.040166, p-adj = 0.00709) and GCS (z = − 3.153009, p-adj = 0.00485) when compared to M-CTCs. Intriguingly, Hoechst positive cells also demonostrated higher FCS (z = − 2.655214, p-adj = 0.0238) and GCS (z = − 2.550165, p-adj = 0.0323) values when compared to M-CTCs, but no significant difference was detected when compared to E-CTCs.

CNAs profiles at arm level depict a heterogenous landscape (Fig. 3D,E). While the majority of isolated cells did not demonostrate relevant CNAs and thus, could be considered as wild-type, we identified CNAs in 3 of 5 E-CTCs (60%), 9 of 34 Hoechst positive cells (26%), and only 1 of 29 M-CTCs (3%). The single CNA-positive M-CTC showed profound aberrations. In contrast, the CNA patterns in Hoechst positive cells were predominantly diffuse, low-level gains/losses. Two of the CNA-positive E-CTCs shared this diffuse pattern, while the third also harboured a medium-level loss on chromosome 21q. No recurrent CNA patterns were observed across the cells.

Collectively, these results highlight that E-CTCs possess a more complex genome consistent with a neoplastic phenotype. However, this genomic instability is not exclusively linked to epithelial markers, as Hoechst positive cells surprisingly showed similar genomic complexity. On contrast, M-CTCs were characterized by a largely stable genome, with a notable absence of copy number abnormalities.

## Discussion

Osteosarcoma remains a formidable challenge in pediatric oncology, with lung metastasis and recurrent disease being major drivers of poor outcomes despite intensified treatment strategies^21^. A critical barrier to improving patient stratification and treatment efficacy lies in the limitations of conventional imaging for detecting low-burden metastatic disease^21^. Therefore, there is a pressing need to develop novel methods for monitoring metastatic progression, particularly the emergence of micrometastases. In this context, the analysis of CTCs emerges as a promising liquid biopsy approach to address this diagnostic gap and enable earlier detection and improved patient stratification. Our study addresses this unmet need by developing and validating a robust pipeline for the isolation, genomic characterization, and clinical correlation of CTCs in pediatric OS patients for the first time using this methodology.

In the context of OS, CTCs were often defined in relation to their phenotypic characteristics^22^ and the presence of circulating elements is often claimed as tumoral based solely on morphological features or marker-dependent enrichment, without downstream genomic verification.

A cornerstone of our work is the genomic validation of CTCs from OS patients, which was first established by a proof-of-concept genomic analysis confirming the neoplastic origin of the isolated BCTC population. For the first time, we performed WES directly on BCTCs (pooled CTCs population) from a metastatic patient (OS4) without prior WGA, which potentially introduces artifacts. This analysis demonstrated a shared mutational landscape and CNAs profile between BCTCs, the primary tumor, and metastatic lesions. This direct genomic concordance, exemplified by shared somatic variations in genes such as AP1M2, KIF1A, SGCG, and SLC16A3 provides a preliminary evidence that BCTCs are indeed tumor-derived and could represent a clonal reflection of the patient’s disease. Notably, among the mutated genes identified, SLC16A3 (MCT4) stands out for its potential relevance. MCT4 is a key monocarboxylate transporter responsible for lactate efflux and is frequently upregulated in hypoxic tumors, where it facilitates metabolic reprogramming and supports cancer cell survival^23^. Mutations in this gene may disrupt lactate export, possibly enhancing the Warburg effect and contributing to the aggressive metabolic phenotype of OS^24^. Mutations were also observed in AP1M2, a gene encoding a subunit of the adaptor protein complex AP-1, which regulates protein sorting between the trans-Golgi network and endosomes^25^. In addition, KIF1A, a kinesin motor protein, may contribute to altered intracellular transport and has been associated with tumor cell motility in other cancers^26^. SGCG, part of the sarcoglycan complex, plays a role in membrane stability and may influence cell–matrix interactions, with possible implications for invasion and metastasis^27^. While the functional impact of these mutations remains to be elucidated, they may represent novel contributors to OS pathobiology.

The mesenchymal nature of OS has historically complicated CTCs identification, as standard epithelial markers like EpCAM, commonly used in other solid tumors (such as lung or breast carcinomas)^28,29^, are often absent^30^. Our approach, combining physical enrichment with a subsequently marker-dependent selection for both E-CTCs and M-CTCs phenotypes, allowed us to capture the phenotypic heterogeneity of CTCs in OS. While M-CTCs were more abundant mirroring the mesenchymal origin of OS, we successfully identified E-CTCs, aligning with previous observations of epithelial-like CTCs in OS patients^19^. This supports the concept of phenotypic plasticity in OS, potentially involving mesenchymal-to-epithelial transition (MET)^31^, which could be a response to the tumor microenvironment or systemic therapy. Concerning the single-cell analyses performed on OS patient-derived CTCs, our CNAs analysis further elucidated the genomic characteristics of isolated CTC subtypes and Hoechst positive cells. The fact that M-CTCs could not be reliably distinguished based on CNA profiles in group analysis suggests a greater genomic similarity to non-tumoral circulating cells or a higher degree of genomic stability in this phenotype, or simply that the specific CNAs measured were not sufficiently discriminatory for M-CTCs. Interestingly, a similar observation was reported in a study on non-small cell lung cancer (NSCLC)^32^, where a subset of cells positive to vimentin (Vim+), a widely used mesenchymal marker to identify M-CTCs, did not exhibit CNAs and were classified as non-tumorigenic mesenchymal circulating cells, displaying a “flat” CNAs profile comparable to that of control leukocytes. This suggested that not all Vim+ cells were necessarily malignant, and some might have shared genomic features with normal circulating cell populations. Moreover, several reports have highlighted that vimentin is also expressed in other non-tumoral circulating cell types, including endothelial cells, hematopoietic-derived mesenchymal cells, and reactive stromal cells^33–35^. This lack of specificity poses a significant limitation in the accurate identification of M-CTCs based solely on vimentin expression. Consequently, further genomic analyses, such as CNA profiling, are necessary to distinguish true tumor-derived M-CTCs from normal counterparts. In light of these findings, it becomes evident that vimentin may not be an optimal marker for identifying M-CTCs also in OS^19,36^.

Despite lacking statistical significance, the distribution of ΔE-CTC values and the elevated Spearman’s rho indicate a possible correlation with histological necrosis that should be explored with a larger sample size. Specifically, the GR patient (with the higher degree of histological necrosis) showed an increase in E-CTCs after treatment, while PR patients more frequently exhibited a decrease. This finding, exemplified by patient OS17 (GR, with increased E-CTCs) and OS12 (PR, with decreased E-CTCs and chemo-refractory disease), highlights a potential discordance between local tumor response and the dynamics of circulating elements. This apparent discrepancy may reflect the complex biology of OS: while histological necrosis is a marker of local cytotoxic efficacy^37^, CTCs, particularly those with epithelial characteristics, may represent residual systemic disease activity, or even treatment-induced mobilization of tumor cells into circulation. Notably, Chalopin et al*.*^14^ showed that ifosfamide increased the number of CTCs during early tumor development while simultaneously reducing pulmonary tumor nodules. In this last view, circulating tumoral elements could be interpreted as an ephyphenomenon of the primary mass dynamic and one possible explanation for this unexpected observation is that effective chemotherapy may induce phenotypic plasticity (in particular mesenchymal-to-ephitelial transition) in OS cells, making them more detectable as E-CTCs^20,38^. Alternatively, extensive tumor cell death in the primary site might lead to release of tumor cells into the bloodstream, some of which may acquire epithelial features as a result of therapy-induced plasticity and mechanical shedding^39,40^. These findings support the need for a more integrated approach to treatment evaluation, combining established metrics with optimized liquid biopsy-based biomarkers to gain deeper insight into OS biology and therapy response.

Our study has limitations, primarily related to the small sample size of the patient cohort (n = 6 OS patients). This limits the statistical power for broader correlations and generalizability of our observations, particularly related to the clinical significance of E-CTC dynamics. Furthermore, for this reasons, survival outcomes should be considered as descriptive and no correlatons could be hypotesize with CTCs enumeration. Future studies with larger patient cohorts are essential to validate these findings, establish robust prognostic or predictive values for E-CTC counts and specific CNAs, and explore the biological mechanisms underpinning the observed correlation between E-CTCs and histological necrosis.

In conclusion, our study validates a robust approach for isolating and genomically characterizing CTCs in pediatric OS, confirming their tumor origin and potential as dynamic biomarkers. The identification of E-CTCs and their correlation with disease features underscores their value for monitoring disease progression and guiding personalized therapy. Importantly, CTC-based monitoring provides a universal strategy applicable to all patients, regardless of molecular heterogeneity, as specific mutations may vary between patients and over time within the same individual. By addressing the limitations of this approach and proposing solutions, our work highlights the robustness and broad relevance of CTCs for clinical application. Larger studies are needed to confirm these findings and support their translation into practice.

## Methods

### Study design

The study was part of The Omics SCiences AgaiNst Osteosarcoma (TOSCANO) project, a prospective, noninterventional, non randomized study conducted by the University of Florence, the University of Pisa and the Fondazione Pisana per la Scienza ONLUS. The study was approved by the regional ethical committee (Comitato Etico Regione Toscana—Area Vasta Nord Ovest) on 10/09/2020 (approval number 18030) and conducted according with the declaration of Helsinky and relevant guidelines and regulations. Informed consent was obtained from parents or legal guardians and/or patients as appropriate at the time of admission for initial diagnosis. Biological material was pseudo-anonymized in accordance with insitutional guidelines. Patient demographics, tumor characteristics, histological findings, imaging results (including maximum standard uptake values [SUVmax] from PET scans at various time points), and pathological data were collected. The project was founded by “Bando Ricerca Salute 2018” from the Tuscany region (approval decree n. 975 on 16/01/2020). This study included children and adolescents and young adult (AYAs) patients until age 21 diagnosed with high-grade OS enrolled in a treatment protocol consisting of neo-adjuvant chemotherapy with Methotrexate, Adryamicin, Cisplatin backbone (MAP), local treatment and adjuvant chemotherapy (MAP or high-dose ifosfamide as per treatment protocol), irrespective of their histological subtype between 2020 and 2023. As per standard practice, we defined the response to neoadjuvant chemotherapy by the degree of histological necrosis in the FFPE surgical specimens. According to established clinical protocols, patients were categorized as good responders (GR) or poor responders (PR) based on whether tumor necrosis was ≥ 90% or < 90%, respectively^41^. Finally, healthy control participants were enrolled by U.O. Ortopedia 1 AOUP following the acquisition of informed consent.

### Human osteosarcoma tissues and blood collection

Five milliliters of blood was collected in EDTA tubes for CTCs detection at the time of the diagnostic biopsy (pre-treatment, named PRE) and immediately before the surgical treatment (thus after the neoadjuvant chemotherapy, named POS). Blood was obtained from peripheral veins and only the tubes beyond the third were considered in order to minimize the risk of contamination from exogenous epithelial elements. Peripheral blood mononuclear cells (PBMCs) were obtained from whole blood through density gradient centrifugation (Ficoll Paque GE17-1440-02, Sigma-Aldrich) as the standard procedure. The pellet was frozen vital with 90% of FBS and 10% of DMSO and stored at − 140 °C.

Formalin-fixed paraffin-embedded (FFPE) of OS4 diagnostic biopsy both pre-treatment and from the metastatic site were collected.

### CTCs enrichment and isolation

#### CTCs enrichment from vital PBMCs

CTCs were enriched using Parsortix Cell Separation System (Angle plc, Surrey, UK). PBMCs were thawed and, after a PBS wash, resuspended in 9 ml PBS in a tube BD vacutainer and run in a 6.5 µm separation cassette following the manufacturer’s instructions.

#### Whole exome analysis

In one case (OS4), we carried out whole-exome sequencing (WES) on the primary tumor, the metastatic lesion, and the BCTCs enriched from the patient’s blood following the Parsortix separation. Our goal was to track the presence of mutations and assess whether the enriched CTCs truly originated from the tumor by comparing their mutational profiles with those of both the primary and metastatic sites. Therefore, at the time of surgery, two identical 5 mL blood samples were collected from the patient. One tube was used for CTC enumeration, while the other was processed for whole-exome sequencing (WES) of the BCTCs. Genomic DNA was extracted from the original FFPE tissue sample (both primary and metastasis) using Maxwell 16 Tissue LEV DNA Purification Kit (Promega, Madison, WI, USA), following the manufacturer’s instructions. DNA from BCTCs was extracted using Maxwell 16 Cell LEV DNA Purification Kit (Promega, Madison, WI, USA), following the manufacturer’s instructions, and was used directly without amplification. Whole-exome library preparation was performed using Illumina DNA Prep with Exome 2.5 Enrichment (Illumina, San Diego, CA, USA), following manufacturer’s procedure. Paired-end sequencing was performed using NextSeq 2000 (Illumina, San Diego, CA, USA) with 151 bp of read length loading on NextSeq 1000/2000 P2 Reagents (200 Cycles) v3. WES was also performed on an additional blood sample from the patient collected at the time of the diagnosis to subtract germline mutations.

The variants were annotated using Cancer-Related Analysis of Variants Toolkit (CRAVAT)^42^ after filtering for germline variants identified by the exome analysis performed on blood. Additonal filtering for somatic variants excluded common artifacts of variant claling such as “strand bias”, “panel of normal” and “normal artifact”. We therefore included somatic exonic (except for synonymous variants) or splice site variants with an allelic frequency ≥ 2.0% and a total read depth ≥ 20. Additionally, manual inspection with Integrative Genomics Viewer (IGV)^43^ was performed to confirm the presence of the variations and the absence of additional artifacts.

#### Immunofluorescence of single-cell suspensions

The cell suspensions obtained after Parsortix enrichment were transferred in 1.5 ml LoBind tubes and washed three times with DPBS. The cells were fixed adding 400 µl of Paraformaldehyde 4%. The antibodies chosen for the staining were : anti-CD326 FITC (EpCAM) (11-5791-82, Invitrogen, Thermo Fisher Scientific, Waltham, MA, USA), anti-Cytokeratin 8 FITC (11-9938-82, Invitrogen, Thermo Fisher Scientific, Waltham, MA, USA), anti-Cytokeratin 18 FITC (MA1-10326, Invitrogen, Thermo Fisher Scientific, Waltham, MA, USA) and anti- Cytokeratin 19 FITC (MA5-28576, Invitrogen, Thermo Fisher Scientific, Waltham, MA, USA) for epithelial cells (E-CTCs) and anti-Vimentin APC (MA5-28601, Invitrogen, Thermo Fisher Scientific, Waltham, MA, USA) and anti-TWIST1 APC (PA5-46902, Invitrogen, Thermo Fisher Scientific, Waltham, MA, USA) for mesenchymal cells (M-CTCs) anti-CD45 PE (12-0451-82, Invitrogen, Thermo Fisher Scientific, Waltham, MA, USA) as a negative control, and Hoechst 33342 (62249, Thermo Fisher Scientific, Waltham, MA, USA) for nuclei (DAPI cells). CD45, a common marker for the hematopoietic lineage, was used as negative control.

#### Single cell Isolation by DEPArray NxT

Single cells were isolated and sorted with DEPArray NxT (Menarini, Silicon Biosystems, Bologna, Italy). After the immunofluorescence of the CTCs suspensions, the samples were washed two times with 1 ml of SB115 Buffer (Menarini, Silicon Biosystems) and the cells were loaded on the DEPArray NxT cartridge following the protocol instructions. CellBrowser analysis software, integrated into the DEPArray system, allows to view and to select cells from the particle database according to multiple criteria, based on qualitative and quantitative marker evaluation and cell morphology. This software enables to create populations and sub-populations of cells using some analysis tools as scatter plots, histograms, and image panels. Cells become unroutable based on their positions, when these are out of the cage it is no longer possible to move them and therefore complete the recovery. Before acquisition, compensation was applied through the integrated fluorophore wizard in order to minimize the spillover between channels, by adjusting exposure, gain and lamp intensity for each channel and accepting a maximum spillover of 5%. Initial acquisition parameters and detail on the workflow are presented in the supplementary material. According to standard operating procedures of the DEPArray platform, morphological parameters should be adjusted per-experiment while we applied a signal intensity treshhold of 50 to define the positivity for a specific marker. However, since the instrument relies on an operator-dependent validation trough a direct visualization of the single cell and the labeling, all the the events were manually inspected from an trained operator in order to evaluate also the correct distribution of the fluorence signal within the defined cells. We therefore excluded clusters of two or three cells, clumps, and spurious events and focused only on single cells with the desired fluorescence analyzing only the “centered” DAPI cells in the cage and, ultimately, only true single cells were categhorized and selected based on fluorescence labeling and morphology. Afterwards, about 20 different single cells were recovered for each tumor patient and volume reduction was performed with VRNxT-Volume Reduction Instrument (Menarini, Silicon Biosystems) according to the instruction manual. The isolated cells were stored at − 20 °C until later downstream analyses.

#### Low pass analysis on CTCs

Whole-genome amplification and Low Pass analysis on all recovered CTCs was performed using the SMARTer Picoplex Gold Single Cell DNA-Seq kit (Takara Bio, Muntain View, USA) following the manufacturer’s instructions to detect chromosomal aneuploidies and CNAs with a low sequencing depth. To sequence our libraries, we used NextSeq 2000 (Illumina, San Diego, CA, USA) with 151 bp of read length loading on NextSeq 1000/2000 P1 Reagents (300 Cycles) v3 for 96 cells per run.

#### CNAs call and analysis

For whole-exome sequencing, Copy Number Aberrations (CNAs) were estimated from BAM files using CNVkit (Talevich et al.). In parallel, data from Low-Pass Whole Genome Sequencing were processed with IchorCNA, a tool optimized for low-coverage (~ 0.1x) data which employs a probabilistic model to segment the genome and predict CNAs. The outputs from both pipelines were subsequently analyzed using the CNApp tool^43^. We then filtered IchorCNA estimation according to the GC-Map correction MAD, filtering out values > 0.2. Filtered data processed by IchorCNA required resegmentation within CNApp. This was necessary because the IchorCNA pipeline analyzes each sample in isolation, making it unable to correct for technical noise that becomes apparent when looking at a whole dataset. By applying the resegmentation algorithm of CNApp to the entire cohort of CTC samples, we performed a crucial cohort-level normalization since it redefine CNA boundaries and adjust sample-specific copy number thresholds. This process reduces noise across all samples, allowing for a more accurate and reliable identification of genuine CNAs. In contrast, the output matrix from CNVkit was analyzed directly in CNApp without further resegmentation.

Following the analysis in CNApp, the resulting TSV files were processed in R Statistical software (v4.3, R Core Team 2025) to generate final heatmaps (tidyverse, ComplexHeatmap, circlize, grid, RColorBrewer, gtools). For a rigorous interpretation, CNAs among CTCs were classified based on the copy number log₂ ratio (logR). A region was defined as a gain if its logR was greater than 0.2, a loss if less than − 0.2, and neutral if it fell between these two thresholds. Gains were further stratified into three categories: low-level for logR values between 0.2 and 0.58, medium-level for values between 0.58 and 1, and high-level for values greater than 1. Similarly, losses were categorized as low-level for logR values between − 0.2 and − 1, medium-level between − 1 and − 1.75, and high-level for values less than − 1.75.

### Statistical analysis

Statistical analysis was performed with GraphPad Prism 9.0 for Windows (BOSon, Massachusetts USA), R Statistical Software (v4.3, R Core Team 2025), NCSS (NCSS 2023 Statistical Software. NCSS, LLC. Kaysville, Utah, USA) and with in-built applications of CNApp portal for copy number alterations integrative analysis (https://github.com/ait5/CNApp). The Wilcoxon signed-rank test was applied for paired data as appropriate. We explored correlations between the difference in CTC counts after neoadjuvant chemotherapy and at diagnosis (∆CTCs) and relevant clinical variables using a two-tailed Spearman’s rho statistic. This was also stratified for E-CTCs and M-CTCs. The clinical variables included metastases or lung nodules at diagnosis, histological necrosis, SUVmax at presentation (T0), at pre-surgical reassessment (T1) and the difference in SUVmax between SUVmax at T1 and T0 (∆SUVmax).

To compare CNA profiles among different CTC groups, we evaluated three key metrics derived from CNApp: the broad (BCS), focal (FCS), and global (GCS) levels of genomic imbalance^44^. Notably, CNA scores were calculated after the resegmentation process as previously described. We then evaluated differences in BCS, FCS, and GCS across E-CTCs, M-CTCs, and Hoechst positive cells using the Kruskal–Wallis test and Dunn’s post-hoc comparisons with Bonferroni correction. A *p* value of < 0.05 was considered significant for all statistical tests.

Lastly, for descriptive purposes, survival analysis was performed using Kaplan–Meier estimates.

## Supplementary Information

Below is the link to the electronic supplementary material.

Supplementary Material 1

Supplementary Material 2

Supplementary Material 3

Supplementary Material 4

Supplementary Material 5

Supplementary Material 6

## Data Availability

Raw FastQ files from low-pass sequencing are publicly available in the EMBL-EBI ArrayExpress database (accession no. E-MTAB-16563).
