# Supplementary material for: Decoding CTCs in osteosarcoma: the molecular journey from initial tumor to metastasis
Source: Sci Rep. 2026 Apr 3;16:15838. doi: 10.1038/s41598-026-47094-5 (PMC13194890; doi:10.1038/s41598-026-47094-5)
Supplement: Supplementary file 6 — Supplementary Material 6 [file 41598_2026_47094_MOESM6_ESM.docx]

**Supplementary material**

**Patients and clinical course**

Patient’s characteristic are presented in the table below. According to treatment protocols, patients have received 2 courses of MAP chemotherapy before the surgery composed by methotrexate 12 g/mq week 0,1,5 and 6, and doxorubicin 75 mg/mq plus cisplatin 120 mg/mq on week 2 and 7. No amputations were performed, but all patients had benefit of conservative surgery at U.O. Ortopedia 1 AOUP performed by highly-specialized surgeons. Adjuvant treatment was selected in relation to the treatment protocol. Two PR patients received high-dose ifosfamide (3 gr/mq/die for days 1 to 5) as adjuvant treatment according to ISG/AIEOP 2021 recommendations, while other PR Pgp-negative patients (n=3) enrolled in ISG/AIEOP OS2 protocol followed a MAP backbone throughout the adjuvant phase. As per center choice, all PR were treated with Mifamurtide (2 mg/mq for 2 times per week for 3 months, than weekly for 6 months).

Table S1. Patient clinicopathological characteristics. Lung metastasis was defined as present (1) if the clinical criteria outlined in the main text were met. The "Additional Nodules" column was marked as present (1) if the patient had either confirmed lung metastases or other lung nodules that did not meet the clinical criteria for metastasis. The total count of all lung nodules is specified in the subsequent "Number" column. Histological necrosis is expressed as a decimal proportion, where 1.0 is equivalent to 100% (e.g., a value of 0.8 represents 80%). PR and GR are defined according to the main text. SUVmax(T0) represent the values obtained by 18FDG-PET on the primary tumor at the diagnosis, while SUVmax(T1) was acquired before the surgery after the neo-adjuvant chemotherapy. F: female; GR: good responders; HD-IFO: high-dose ifosfamide; L: left; M: male; MAP: Methotrexate, Adriamycin and Cisplatin; PR: poor responders; R: right.

To note, best response classified as non-evidence of disease (NED) was observed in all the patients with the exception of OS12 in which a stable-disease (SD) was the best response observed. While only one patient was lost due to a disease progression, all patients except one have experienced another event after the best response (local relapse or lung metastasis). Survival curves and estimates are reported in figure S1. Due to the limited sample size, it was not possible to investigate correlations between survival parameters and CTCs enumeration.


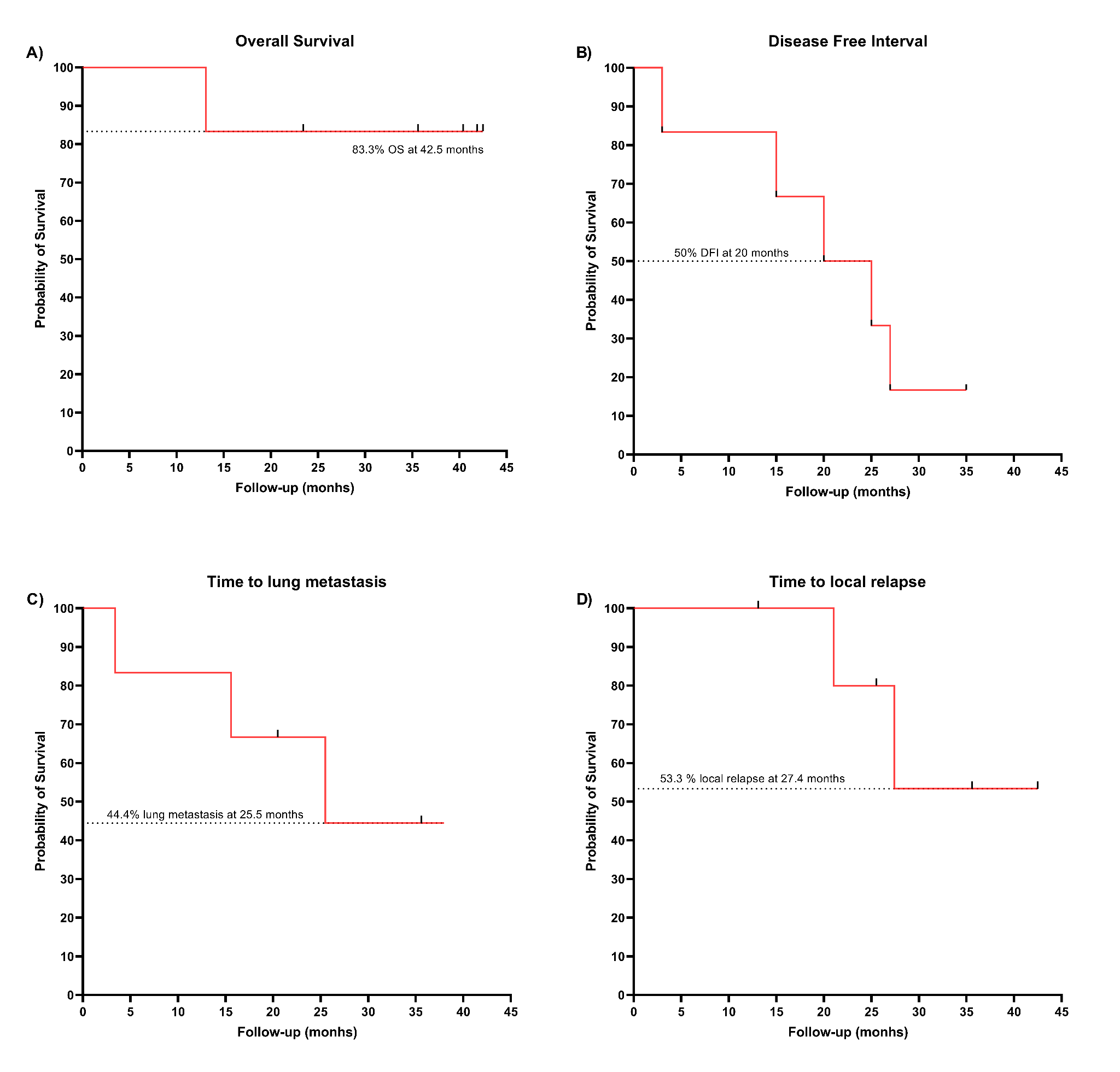


Figure S1. Survival analysis. A) Overall survival was stable from 13.1 months at 83.3% (SE=0.15, at risk=6, events=1). The event was related to disease progression. B) Disease free interval was assessed from the time of the surgery and the first event after the surgery. It reaches the median at 20 months after the surgery (SE=0.20, at risk=4, events=3) and decreases to 16.7% at 27.4 months (SE=0.15, at risk=2, events=5). To note, the follow-up period of the DFI is shorter since the interval was defined after the surgical resection. C) Time to lung metastasis was assessed for considering the time from the diagnosis to the onset of a new lung metastasis. By the end of the 41.9 months after the diagnosis, half of the patients have developed a lung involvement with the median reached at 25.5 months (SE=0.22, at risk=3, events=3). D) Time to local relapse was calculated from the diagnosis to the eventual local relapse. 2 out of 6 patients had developed a local relapse with an overall probability of 53.3% at 27.4 months (SE=0.25, at risk=3, events=2). It was not possible to stratify GR/PR patients because of the cohort imbalance (only one GR). DFI: Disease Free Interval, GR: Good Responders, NED: No Evidence of Disease, OS: overall survival, PR: Poor Responders, SE= Standard Error.

**Quantitative genomic metrics of shared variants.**

The table summarizes the sequencing parameters for the mutations shared among the primary osteosarcoma tumor, the metastasis, and the BCTC of patient OS4. For each variant the nucleotide change, total sequencing depth (DP) and Variant Allele Frequency (VAF %) are provided. Note: DP and VAF were derived from automated variant calling. For shared variants that fell below the standard software thresholds due to low-input DNA or subclonal heterogeneity, metrics were obtained through manual curation and read-counting using IGV (Integrative Genomics Viewer). The identification of identical variants across P, M, and BCTC samples was used as a criterion for high-confidence somatic mutation inclusion. P: Primary Tumor; M: Lung Metastasis; B: CTC Pool.

**CTCs enumeration versus clinical parameters**

Here we report all the investigated correlations between CTCs enumeration and clinical relevant outcomes and some are reported in the subsequent figure S2. Only correlation with a potential clinical or biological significance were tested. For pre-treatment (PRE) CTCs counts we investigated correlations between E-CTCs or M-CTCs and SUVmax(T0) (rho=-0.15, p=ns and rho=-0.52, p=ns respectively) , lung metastasis (rho=-0.13, p=ns and rho=0.39, p=ns respectively), lung nodules (rho=-0.1, p=ns and rho=-0.49, p=ns respectively) and the number of lung nodules (rho=0, p=ns and rho=-0.21, p=ns respectively) without proving any significant correlation. Similarly, post-treatment E-CTCs and M-CTCs enumeration was not correlated with nor SUVmax(T1) (rho=-0.22, p=ns and rho=-0.30, p=ns respectively) and histological necrosis (rho=0.45, p=ns and rho=0.1, p=ns respectively). Finally, neither ∆E-CTCs nor ∆M-CTCs showed any significant overall correlation. However, the relationship between ∆E-CTCs and histological necrosis is worth mentioning. Despite not reaching statistical significance, likely due to the limited sample size, the rho value for this pair was particularly indicative. Furthermore, its p-value was lower when compared to other data where the distribution appeared to be random (see Figure S2). The number and calculation of ∆CTCs can be derived from the information in Figure 2 of the main text.


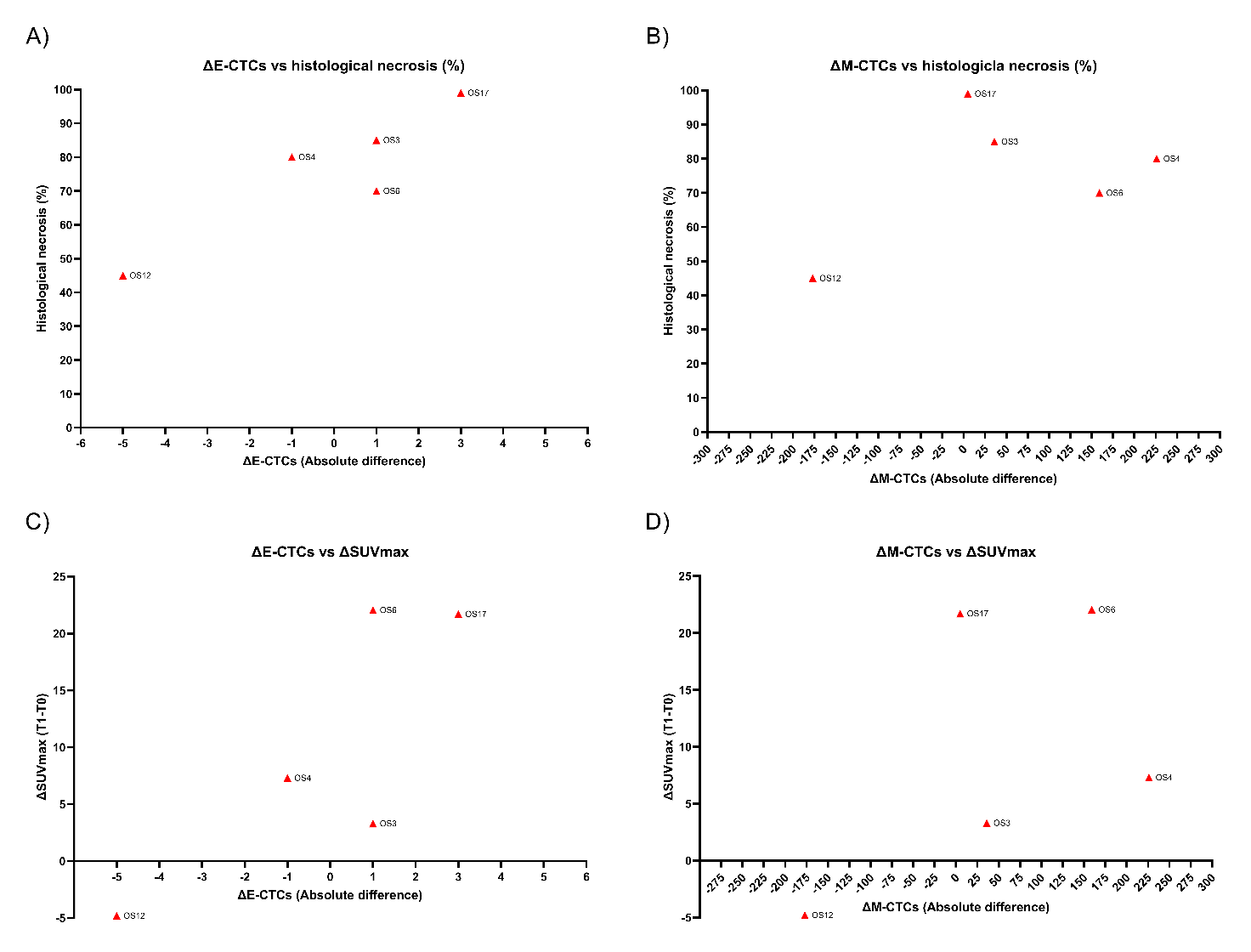


Figure S2. Correlations between ∆E-CTCs and ∆M-CTCs counts and clinicopathological parameters. ∆-CTCs are reported as absolute values. A), C) showed correlations matrices of ∆E-CTCs for histological necrosis and ∆SUVmax that do not reach the statistical significance (rho=0.82, p=0.13 and rho=0.67 p=ns respectively). However, as also shown in the main text, the distribution of values when plotted against histological necrosis could suggest a possible different trend but a larger sample size is needed. Similarly, B) and D) report the same attributes for ∆M-CTCs (rho=0.10, p=ns and rho=0.58, p=ns).

**Overview of the workflow for CTCs isolation and processing**

All the process, form blood sample manipulation to post-processing, is schematically presented in the figure below.


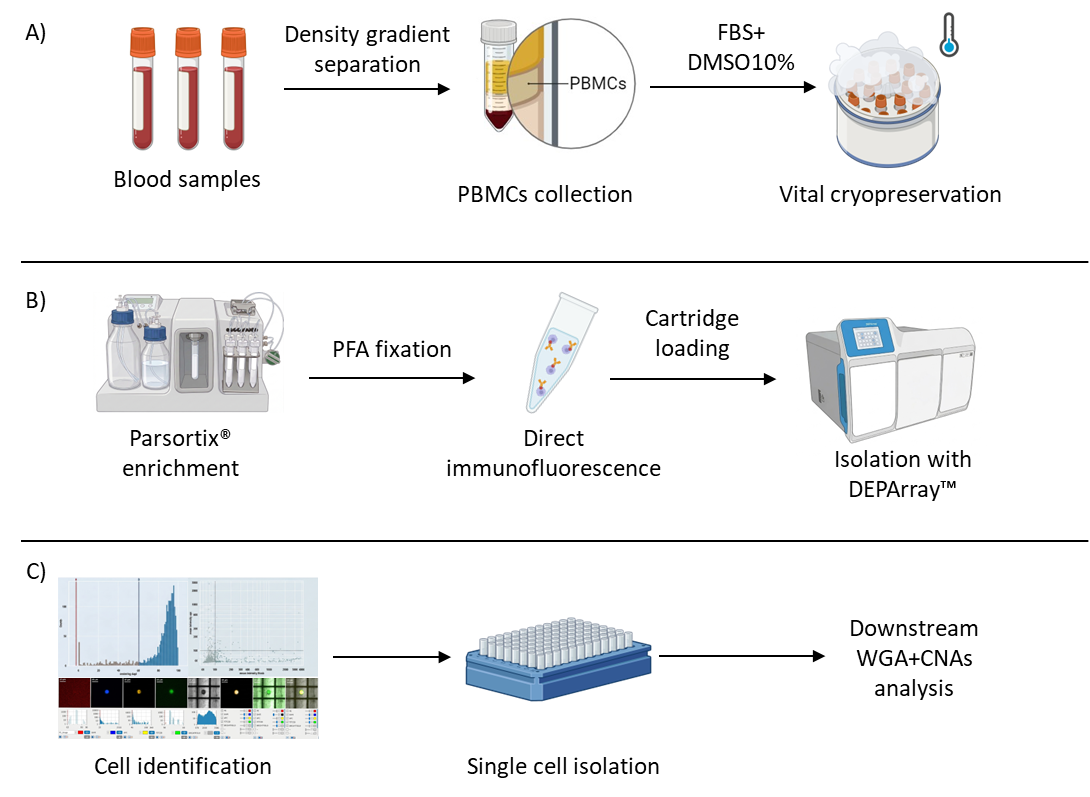


Figure S4 Schematic overview of the CTCs enrichment and isolation. A) Sample collection and processing. Fresh blood sample in EDTA tubes were harvested from patients for an effective processed volume of 5 mL of whole blood. All the samples were processed within at maximum 4 hours form the collection in order to ensure the viability of CTCs. PBMCs were obtained through a density gradient centrifugation with Ficoll Paque (GE17-1440-02, Sigma-Aldrich), then cells were stored vital at 140°C in FBS+DMSO10% in order to preserve their vitality and original morphology. B) After a rapid thawing and a gently PBS washing, products were enriched through the Parsortix system. Then, the BCTCs were fixed with 4% PFA. Direct immunofluorescence (against CD45 as negative control conjugated with PE, CK8/18/19 and EpCAM conjugated with FITC, vimentin and twist conjugated with APC) and Hoechst 33342 were than performed in solution according to standard operating procedures of DEPArray platform. Afterwards, the cartridge was loaded and prepared for the DEPArray isolation. C) DEPArray-based cell selection, single cell isolation and subsequent downstream analysis. Cells were then identified according to their markers expression. Thereby, they were classified as E-CTCs, M-CTCs CD45-positive cells or Hoescht-positive cells when no marker expression was detected. Single cells were subsequently isolated and stored at -20°C. Afterwards, WGA and low-pass CNAs detection was performed with SMARTer Picoplex Gold Single Cell DNA-Seq kit (Takara Bio, Muntain View, USA) and sequenced within the Illumina platform. Please note that the Parsortix (panel A), the DEPArray (panel B) and the first image of the panel C, were manipulated for graphical purposes with Nano Banana 2 (accessed with Google Gemini Pro on 14^th^ March 2026) from an original picture of the authors. Other figures were created with Biorender. BCTCs: bulk CTC populations, PBMCs: Peripheral blood mononuclear cells, WGA: Whole Genome Amplification.

**Experimental set up and selection strategy with the DEPArray platform**

Herein we present the initial experimental setting of our workflows, spillovers were defined trough the integrated DEPArray fluorophore selection wizard tool as recommended. A maximum spillover of 5% was accepted during the set-up phase. Standard operating procedures of the DEPArray platform allowed a minimal change of the exposure time and/or gain after a preliminary acquisition in order to ensure the maximum quality of the final acquisition without dramatically affecting the desired spillover between channels.

|  | | | | Estimated spillover (target channel) | | | |
| --- | --- | --- | --- | --- | --- | --- | --- |
|  | Exposure (ms) | Camera Gain | Lamp intensity | H33342 | PE | FITC | APC |
| H33342 | 80 | 1% | 15% | / | 0% | 0% | 0% |
| PE | 250 | 1% | 100% | 1% | / | 4% | 0% |
| FITC | 350 | 1% | 100% | 1% | 2% | / | 0% |
| APC | 300 | 1% | 100% | 0% | 0% | 1% | / |

Table S2. Spillover estimation and channels setup. The integrated fluorophore selection wizard was used to estimate the spillover between the channels and, according to DEPArray standard operating procedures, a maximum of 5% was tolerated. A minimal change of exposure and gain was allowed after the preliminary acquisition in order to ensure the maximum image quality without affecting the overall spillover between channels (maximum change in gain allowed 2%, maximum change in exposure allowed ±5%). H33342: Hoechst 33342.

After the acquisition, cell selection was performed combing both gating parameters (although not strictly and minimally changed on a sample basis in accordance with the standard procedure of DEPArray platform) and operator visualization. Please note that the trained operator revised all the events in order to ensure that no cell is lost (classified as a spurious event) and the correct classification according to marker distribution. In fact, with the DEPArray platform, the selection process is always supervised and the operator selects the cells of interest by carefully analyzing high-resolution, multi-channel fluorescence images captured for each individual cell trapped within the microfluidic cartridge. However, a sort of “gating” is helpful in making the process more rigorous and reliable.

Cells were automatically assigned as “routable” or “not routable” in dependence of their dimension and position in the dielectrophoretic cage. Subsequent filtering and evaluations were performed on both populations while only routable cells could be recovered for downstream analysis.

First, we identified and excluded spurious events by selecting only events with a centering DAPI parameter ≥60. Afterwards, we distinguished PE positive cells (CD45+) form PE negative cells (CD45-) based on the signal intensity (more or less than 50). Then, PE negative populations were gated according to the mean intensity of FITC (EpCAM+CK8/18/19) versus APC (vimentin + TWIST) by defining four subpopulation with a threshold of 100 on a logarithmic scale. Then, the operator verifies that a nuclear marker (HOECHST 33342) is correctly localized, while a positive target marker (EpCAM plus CK8/18/19 or TWIST plus vimentin) exhibits a distinct distribution with an appropriate signal intensity >50. Simultaneously, the trained operator visually confirm the absence of exclusion markers (CD45) to rule out hematopoietic-derived cells. Globally, we therefore identified different subpopulations:

- E-CTCs: FITC^+^, APC^-^, PE^-^, Hoechst^+^
- M-CTCs: FITC^-^, APC^+^, PE^-^, Hoechst^+^
- E/M-CTCs: FITC^+^, APC^+^, PE^-^, Hoechst^+^
- Hoechst -positive cells: FITC^-^, APC^-^, PE^-^, Hoechst^+^
- Hematopoietic-derived cells (not CTCs): PE^+^, Hoechst^+^ (note: we did not find FITC^+^ cells in this subpopulation)

Hematopoietic-derived cells were excluded from counting and downstream analysis. An example of the gating strategy is shown below.


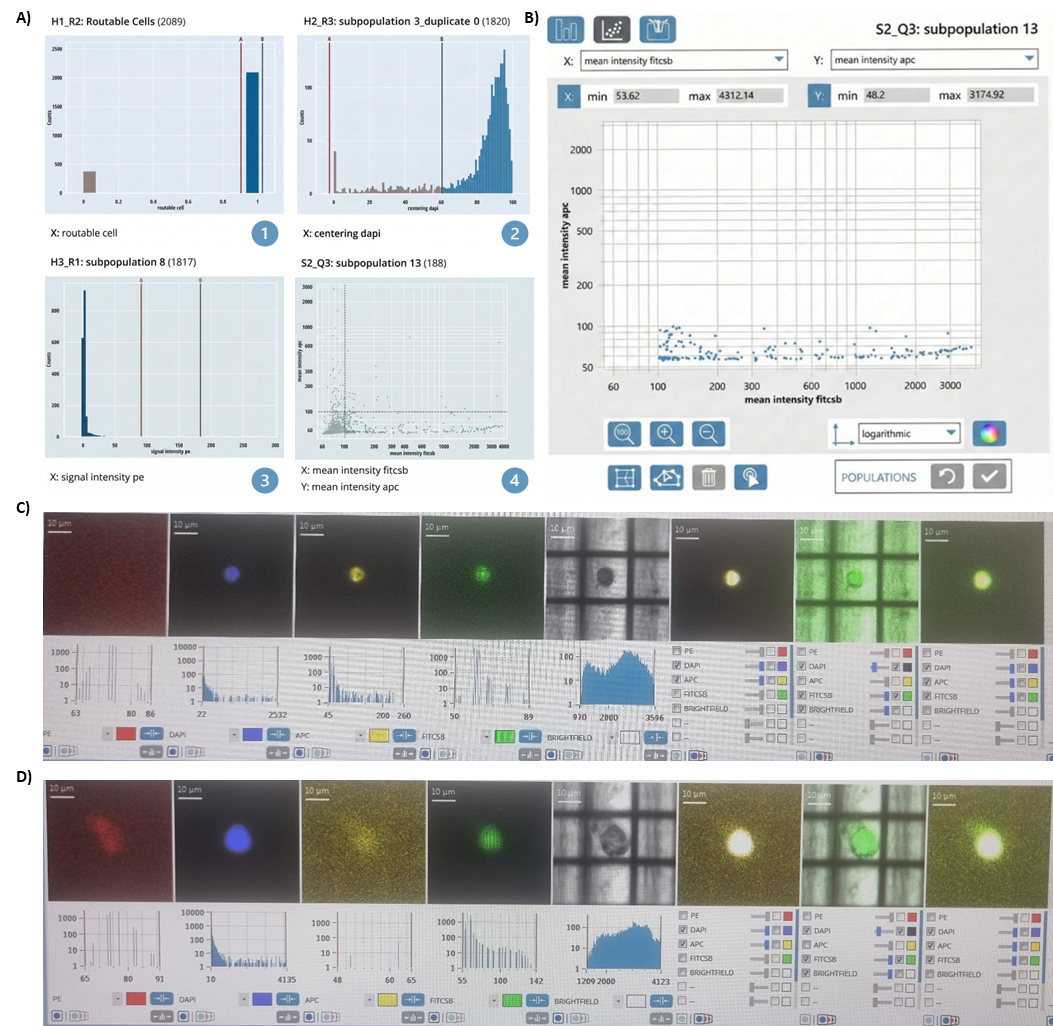


Figure S3. Example of gating strategy and manual selection. A) Overview of the gating strategy for routable E-CTCs (FITC-positive cells). First (1), all routable cells were considered, events with centering DAPI <60 (2) were excluded since they could be appointed as spurious events and not cells. Afterwards, PE-positive cells were excluded (3) and B) cells with a mean intensity on the FITC channel >100 and a mean intensity on the APC channel <100. We therefore identified four subpopulations at this stage (FITC^+^APC^+^, FITC^+^APC^-^, FITC^-^APC^+^, FITC^-^APC^-^) D) Example of an operator-dependent validation of the gated cells. In the first panel an APC-positive (M-CTC) cell is shown while in the second one a FITC-positive (E-CTC) is considered. Brightfield image helps the operator to verify the shape of the cell and its dimensions in combination with the DAPI signal (please note that the channel is named DAPI independently on the use of the compound applied, in this case Hoechst 33342). Beyond the designated signal intensity >50 to define the positivity for a particular marker, the cellular distribution as well as the shape of the emission curve (almost continuous, with a sort of “saddle” on the right side) is essential to correctly assess the positivity for the specific marker. Please note that image A) and B) were post-processed with Nano Banana 2 (accessed with Google Gemini Pro on 10^th^ March 2026) in order to improve the graphical quality, without affecting the presented data. E-CTCs: epithelial circulating tumor cells; M-CTCs: mesenchymal circulating tumor cells.

**CNAs metrics and original data**

Detailed CNAs metrics of FCS, BCS and GCS are reported in the following table.

| CTC phenotype | N | FCS median (IQR) [range] | BCS median (IQR) [range] | GCS median (IQR) [range] |
| --- | --- | --- | --- | --- |
| Epithelial | 5 | 2.00 (8.00) [0.00 - 19.00] | 2.00 (11.00) [0.00 - 13.00] | 1.54 (2.32) [-0.85 - 6.94] |
| Hoechst | 34 | 0.00 (2.75) [0.00 - 22.00] | 0.00 (2.00) [0.00 - 12.00] | -0.85 (1.77) [-0.85 - 6.37] |
| Mesenchymal | 29 | 0.00 (0.00) [0.00 - 8.00] | 0.00 (0.00) [0.00 - 20.00] | -0.85 (0.00) [-0.85 - 6.12] |

Table S3. Metrics of CNAs alterations among different CTCs phenotypes. E-CTCs demonstrated a higher genomic complexity when compared to other M-CTC or Hoechst-positive cells. Non parametric-test results in significative differences but the sample size remains small., E: epithelial, CNAs: copy number alterations CTCs: circulating tumor cells, M:mesenchymal.

Data for CNAs processed by Ichor, grouped and used as input file for CNApp are available in the tab-delimited txt file Input_CNapp_CTC.txt. Processed data with corrected CNAs estimation matrix could be retrieved from the following files: for exome data of OS4 CNA_BY_ARM_EXOME.tsv and CNA_FOCAL_EXOME.tsv (no annotation file needed), while for CTCs data could be retrieved at cna_profile_Arms_by_CTC_TYPE2025-04-09 18_50_59.331676.tsv with the following annotation file Annotation_tracks_by_CTC_TYPE_2025-04-09 18_50_59.330199.tsv. raw data are are publicly available in the EMBL-EBI ArrayExpress database (accession no. E-MTAB-16563).
